# Histological investigation of the impact of streptozotocin-induced experimental diabetes on the healthy gingivae of rats

**DOI:** 10.1080/13102818.2014.943558

**Published:** 2014-10-17

**Authors:** Ahmet Dağ, Ela Tules Fırat, Ersin Uysal, Şennur Ketani, Muzaffer Aydın Ketani

**Affiliations:** ^a^ Faculty of Dentistry, Dicle University, Diyarbakir, Turkey; ^b^ Diyarbakir Vocational School, Dicle University, Diyarbakir, Turkey; ^c^ Faculty of Education, Dicle University, Diyarbakir, Turkey; ^d^ Faculty of Veterinary Medicine, Dicle University, Diyarbakir, Turkey

**Keywords:** experimental diabetes, gingiva of rats, the histological investigation

## Abstract

This study was aimed at the histological investigation of the impact of experimental diabetes on the healthy gingiva of rats. Thirty male Wistar rats were used in this study. The animals were randomly divided into two groups (*n* = 15) prior to the experiment. Group 1 experimental diabetes was created by streptozotocin injection in 15 rats. Group 2 comprised the control group (15 rats). On the 7th, 14th and 21st days after the induction of diabetes by streptozotocin, five animals from each group were euthanized by cardiac puncture. The gingiva of the maxillary left first molar tooth of the sacrificed animals was extracted for histological examination. Histological examination demonstrated that, when compared to the control group, the diabetes group displayed marked hyperkeratosis and parakeratosis of the gingival epithelium on day 21 post-induction. Furthermore, the diabetes group presented with an increased number of inflammatory cells and vasodilatation of the capillaries, in comparison to the controls. The overall evaluation of the findings obtained in this study suggested that diabetes alone could cause changes in the periodontium and affect periodontal health.

## Introduction

Diabetes mellitus (DM) is emerging as a global epidemic, whose complications impact significantly on the quality of life, longevity and healthcare costs. It is estimated that 346 million people currently suffer from diabetes worldwide and the World Health Organization (WHO) predicts that this will increase to 439 million, almost 10% of adults, by 2030.[1]

DM is a metabolic disorder manifested by abnormally high levels of glucose. The hyperglycemic state developed from either a deficiency in insulin secretion or an impaired cellular resistance to the action of insulin is associated with a number of complications, leading to retinopathy, nephropathy, peripheral neuropathy, angiopathy and impaired wound healing.[2,3]

Periodontal disease is vulnerable to the influence of systemic modifiers, acting as risk factors for development and progression of destruction of periodontal tissues. Within several systemic conditions, DM is the most important topic associated with periodontal breakdown.[4]

Periodontal disease is considered as the sixth complication of DM. It has long been observed that diabetic patients have greater tooth loss due to periodontal disease than non-diabetics of comparable age.[5] The severity of the periodontal disease is absolutely influenced by the degree of diabetic status.[6] The phenomenon may be due to the high tissue glucose concentration, as well as due to the presence of metabolic products of the impaired glucose metabolism and it is recognized histologically as enlarged vessel wall with a narrowing of vessel lumen diameter. This process induces disabilities of vessel wall and, in general, leads to abnormal vasculature.[7]

A number of studies found a higher prevalence of periodontal disease among diabetic patients than among healthy controls.[8] In a large cross-sectional study, Grossi et al. [9] showed that diabetic patients were twice as likely as non-diabetic subjects to have attachment loss. Firatli [8] monitored type 1 diabetic patients and healthy controls for five years. The people with diabetes had significantly more clinical attachment loss than controls. In another cross-sectional study, Bridges et al. [10] found that diabetes affected all periodontal parameters, including bleeding scores, probing depths, loss of attachment and missing teeth. In fact, a study has shown that diabetic patients are five times more likely to be partially edentulous than non-diabetic subjects.[11] People with type I and type 2 diabetes appear equally susceptible to periodontal disease and tooth loss.[12]

How diabetes causes predisposition to periodontal disease remains unclear. In the light of new data obtained in most recent research, the association of diabetes with periodontal disease is approached on a scientific basis. To date, the impact of the development of diabetes on the healthy periodontium has not been fully investigated and elucidated. In this study, it was aimed to investigate by histological examination the potential effects of experimentally induced diabetes on the healthy periodontium in rats.

## Materials and methods

A total of 30 adult male Wistar rats (250–300 g) from the Department of Medical Science Application and Research Centre of Dicle University were used. All of the animals were provided with commercial rat chow and water ad libitum and were maintained on a 12-h light/12-h dark cycle, at a temperature of 22 ± 1 °C. The study was performed in accordance with the Helsinki Declaration and with the permission of the Governmental Animal Protection Committee. The animals were randomly divided into two groups (*n* = 15) prior to the experiment. Group 1 irreversible experimental diabetes was created by streptozotocin injection in 15 rats. Group 2 comprised the control group (15 rats). These groups were further divided into three subgroups for sacrifice on the 7th, 14th and 21st days after the induction of diabetes by streptozotocin. From each group of five animals, harvested tissue samples were subjected to histological analyses.

### Induction of experimental diabetes

Prior to the induction of experimental diabetes, the rats were weighed and their body weights were recorded. Blood samples were taken from the caudal vein of each animal, and blood glucose levels were measured using a glucometer. With an aim to induce diabetes, the animals were administered with a single intraperitoneal dose (50 mg/kg) of streptozotocin (STZ, Sigma Chemical Company, St Louis, MO) dissolved in 0.2 mL of citrate buffer (0.1 mol/L, pH = 4.5).[13] Three days after the injection, blood glucose levels were measured for a second time, and a twofold increase was observed. Thus, experimental diabetes was confirmed to have been induced in the animals. Following the induction of diabetes, five rats were sacrificed from each group on days 7, 14 and 21. The upper left first molar tooth's gingiva of the sacrificed animals was extracted for histological examination.

### Histological procedure

**Figure 1.**
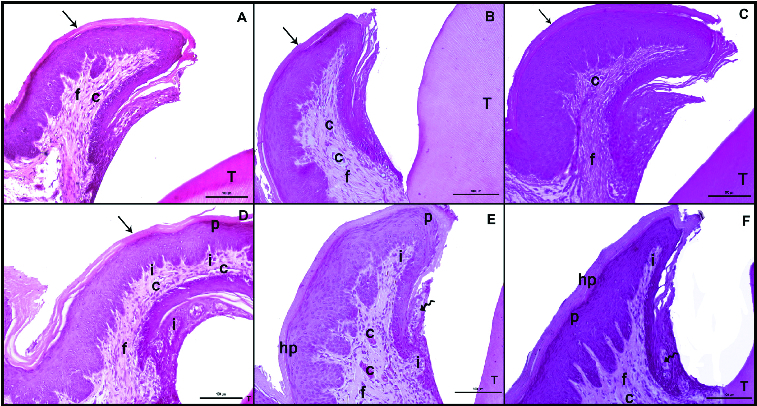
Hematoxylin–eosin (H&E) staining after the induction of diabetes by streptozotocin. Histological images of the non-diabetic (control) rats: on day 7 (A), day 14 (B), day 21 (C); and diabetic rats: on day 7 (D), day 14 (E), day 21 (F). Tooth (T); corneum (arrow), capillary (C), fibroblasts (f), collagen (arrowhead), inflammatuar cells (i), parakeratosis (p), hyperkeratosis (hp), interepthlium inflammatory cell (curved arrow), disorganized structure of collagen fibres (*); scale bar: 50 μm.

**Figure 2.**
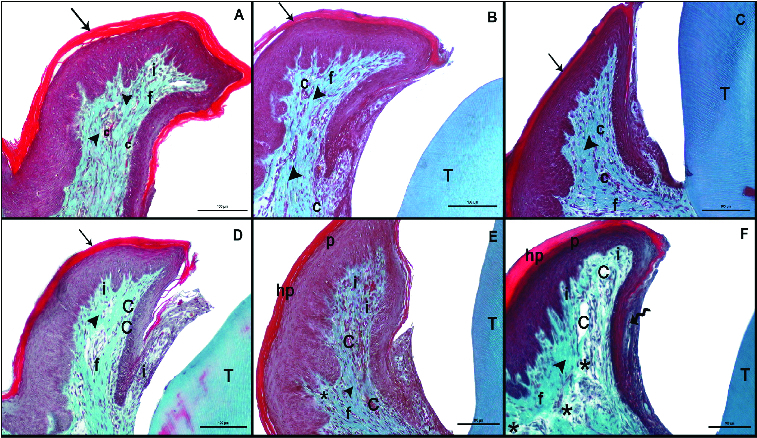
Masson trichrome (MT) staining after the induction of diabetes by streptozotocin. Histological images of the non-diabetic (control) rats: on day 7 (A), day 14 (B), day 21 (C); and diabetic rats: on day 7 (D), day 14 (E), day 21 (F). Tooth (T); corneum (arrow), capillary (C), fibroblasts (f), collagen (arrowhead), inflammatuar cells (i), parakeratosis (p), hyperkeratosis (hp), interepthlium inflammatory cell (curved arrow), disorganized structure of collagen fibres (*); scale bar: 50 μm.

On the 7th, 14th and 21st days after the induction of diabetes by streptozotocin, five animals from each group were euthanized by cardiac puncture under intraperitoneal anaesthesia with ketamine HCI (35 mg/kg) and xylazine (3 mg/kg). Harvested specimens were fixed in 10% formalin for 24 h, decalcified 5% formic acid, dehydrated in graded ethanol baths (100% and 70%), cleared in xylene, embedded in paraffin wax, and serially sectioned at 5 μm. Two sections of each specimens were randomly selected and stained with haematoxylin and eosin (H&E), or Masson's trichrome (MT) for evaluation by light microscopy. H&E was used to evaluate the cellular structures (Figure 1). Connective tissue destruction and vaskuler dilatation were examined with MT (Figure 2). The parameters (inflammation cell, vaskuler dilatation, connective tissue destruction, epithelium hypertrophy, parakeratosis and hyperkeratosis) were scored as follows: 0 = absent, 1 = mild, 2 = moderate and 3 = marked, as previously described by Kirchner et al.[14] To avoid observer bias, the histologist was blinded to the study groups, and the data were recorded with respect to the sample codes. The microphotographs of each preparation were acquired using a Nikon Eclipse-400 digital (Coolpix 4500) camera, which was coupled to a standard research microscope by Kirchner et al. and showed in Table 1.

### Statistical assessment

As the data were not normally distributed, non-parametric statistical analysis methods were used. In order to assess time-dependent differences in the parameters investigated, Friedman's test and the Wilcoxon signed-ranks test were used. For the analysis of categorical data, the chi-square test was performed. *p*-values smaller than 0.05 (*p* < 0.05) were considered to be statistically significant.

## Results and discussion

### Histological results

In the control group, on days 7, 14 and 21, both the keratinized stratified squamous epithelium of the gingiva and the microscopic papillae of the epithelium maintained their normal structure. The gingival epithelium was keratinized and did not display hyperkeratosis or parakeratosis. The sulcular epithelium was also of normal appearance (Figure 1(A)–(C)). The collagen fibres were regular, and the capillaries did not display vasodilatation (Figure 2(A)–(C)).

The comparison of the diabetes group with the control group on days 7, 14 and 21 demonstrated marked hyperkeratosis and parakeratosis in the keratinized stratified squamous epithelium of the gingiva in the diabetes group on day 21. Progressive thickening of the gingival epithelium was observed in the diabetes group between days 7 and 21 (Figure 1(D)–(F)). While the microscopic papillae of the epithelium were regular in the control group, they were observed to be irregular in the diabetes group (Figure 1(E) and 1(F)).

In the diabetes group, the number of inflammatory cells and the vasodilatation of the capillaries were observed to have increased progressively in the connective tissue between days 7 and 21 (Figure 1(D)–(F)). Diabetic rats showed the presence of intraepithelial leukocytes in the sulcular epithelium (Figure 1(F)), which was not observed in the control group. This was also considered as a major finding.

MT staining demonstrated marked hyperkeratosis and parakeratosis of the gingiva in the diabetic group on day 21. This staining also pointed out to the disruption of collagen synthesis and a marked vasodilatation of the capillaries in the diabetes group (Figure 2(D)–(F)).

### Statistical results

The evaluation of the frequency of differences in the parameters investigated, between the control and diabetes groups on day 7, using the chi-square test, demonstrated a statistically significant alteration in the vascular dilatation parameter (*p* = 0.002). Although numerical differences were also detected for other parameters, these were statistically insignificant.

The evaluation of the frequency of differences in the parameters investigated, between the control and diabetes groups on day 14, using the chi-square test, demonstrated statistically significant differences to have occurred in all the parameters, excluding hyperkeratosis. Statistically significant differences were detected for parakeratosis (*p* = 0.002), epithelial hypertrophy (*p* = 0.002), number of inflammatory cells (*p* = 0.036), vascular dilatation (*p* = 0.007), and breakdown of the connective tissue (*p* = 0.002).

The evaluation of the frequency of differences in the parameters investigated, between the control and diabetes groups on day 21, using the chi-square test, demonstrated significant differences for all parameters, including hyperkeratosis (*p* = 0.036), parakeratosis (*p* = 0.010), epithelial hypertrophy (*p* = 0.07), number of inflammatory cells (*p* = 0.019), vascular dilatation (*p* = 0.07) and breakdown of the connective tissue (*p* = 0.019).

The in-group comparison of the rats included in the diabetes group on days 7, 14 and 21 using Friedman's test demonstrated statistically significant differences to exist for parakeratosis, epithelial hypertrophy and vascular dilatation. Statistically significant differences were detected for parakeratosis between days 7 and 14 (*p* = 0.025), and days 7 and 21 (*p* = 0.046). Similarly, epithelial hypertrophy was observed to differ significantly between days 7 and 14 (*p* = 0.025) and days 7 and 21 (*p* = 0.034). On the other hand, vascular dilatation differed significantly between days 7 and 21 (*p* = 0.046). Although numerical differences were also detected for the other parameters investigated, these differences were statistically insignificant Table 2.

**Table 1.** Histological parameters used to evaluate diabetes.

Groups						
	Control group			Diabetes group		
Parameters	7th day (*n* = 5)	14th day (*n* = 5)	21st day (*n* = 5)	7th day (*n* = 5)	14th day (*n* = 5)	21st day (*n* = 5)
Inflammation cells	1,0,1,0,0	1,1,0,1,0	1,0,1,1,0	2,2,2,1,1	2,2,2,2,2	3,3,2,2,2
Vascular dilatation	0,0,0,0,0	0,0,0,0,0	0,0,0,0,0	0,0,1,0,0	2,2,2,1,1	2,2,3,2,2
Epithelium hypertropy	0,0,0,0,0	0,0,0,0,0	0,0,0,0,0	0,1,0,0,0	1,1,1,0,0	1,2,1,0,1
Hyperkeratosis	0,0,0,0,0	0,0,0,0,0	0,0,0,0,0	0,0,0,0,0	0,0,1,1,1	1,1,1,1,1
Parakeratosis	0,0,0,0,0	0,0,0,0,0	0,0,0,0,0	1,1,1,0,0	2,2,2,1,1	2,2,2,1,1
Disorganizes the structure of conjunctive fibres	0,0,0,0,0	0,0,0,0,0	0,0,0,0,0	0,0,0,0,0	1,0,0,1,0	2,1,1,2,1

Note: Scoring: 0 = absent; 1 = mild; 2 = moderate; 3 = marked.

**Table 2.** Friedman's test results obtained for the rats included in the diabetes group on days 7, 14 and 21.

	Mean rank for days				
Diabetes group parameters	7th day	14th day	21st day	Chi-square	*p*
Hyperkeratosis	1.50	1.80	2.70	5.200	0.074
Parakeratosis	1.10	2.60	2.30	8.400	**0**.**015**
Epithelium hypertropy	1.00	2.40	2.60	9.500	**0**.**009**
Inflammation cell	1.50	2.00	2.50	3.846	0.146
Vasculer dilatation	1.30	2.50	2.20	6.500	**0**.**039**
Disorganizesthe structure of conjunctive fibres	1.40	2.00	2.60	6.000	0.050

Bold values indicate *p* < 0.05.

Due to the continuously increasing number of diabetes patients worldwide, as a result of technological developments, increased life expectancy and some other factors, an increased amount of research has been conducted on diabetes and related complications. This study addresses a major concern: how diabetes creates predisposition to periodontal disease.

In the light of new data acquired from recent research, the association of diabetes with periodontal disease is approached on a scientific basis. While diabetes is considered as a risk factor for periodontal disease, literature reports also indicate periodontal disease as a complication of diabetes.[15] In view of this intricate cause and effect relation, the alterations caused by diabetes in the host response and the role of these alterations in the development of periodontal disease constitute a separate field of research, which continues to attract attention.

Although literature reports exist which suggest that diabetes aggravates inflammation in the gingival tissue in individuals already suffering from periodontitis [16,17], to date, the impact of diabetes on the healthy periodontium has not been fully investigated and elucidated. For this reason, the present study was aimed at investigating whether diabetes alone may cause periodontal inflammation or any further alterations in the gingival tissue.

In the present study, diabetes was induced in the rats by the administration of streptozotocin. It was preferred to induce experimental diabetes because clinical trials on the correlation between diabetes and periodontal disease have several limitations related to metabolic control, genetic background, onset and duration of the disease, ethical problems and logistic reasons.[18] Therefore, animal models can aid in answering unsolved questions. Mouse models of experimental diabetes are advantageous in that they have a short generation time, and are easily handled and relatively less costly than other species models.[19]

In the present study, inflammatory cell infiltration of the gingiva was used as a criterion to assess periodontal inflammation. The data obtained demonstrated that the level of inflammatory cell infiltration was higher in the diabetes group, in comparison to the control group. Furthermore, the presence of intraepithelial leukocytes was observed in the sulcular epithelium in the diabetes group, which was not the case in the control group (Figure 1(F)). This was considered as a major finding. Periodontal disease begins with the change of the microbiota in the sulcular region. The intraepithelial infiltration of leukocytes was considered to have resulted from such a change in the microorganisms in this region. Salvi et al. [17] reported that the tissue response to inflammatory stimuli may develop earlier in diabetic individuals than in non-diabetic individuals, which is in support of our findings.

Previous research has shown that, in cases of periodontal disease, diabetes increases vascularization and congestion, which are both signs of inflammation.[20,21] In the present study, following the induction of diabetes, vascularization and vasodilatation were observed also in healthy tissues. These findings comply with those reported in previous investigations.

In 2008, Silva et al. [22] determined that interstitial space increased in the gingival epithelium, in the event of diabetes. In this study, when compared to the controls, the diabetic animals presented with epithelial hypertrophy. This finding was in parallel with the findings reported by Silva et al.

Silva et al. also reported a decrease in the collagen structure and disruption of the collagen fibril structure in the connective tissue of diabetic individuals. Similar to these findings, the present study also demonstrated an irregularity and decrease in collagen fibres. On day 21 following the induction of diabetes, the microscopic papillae of the epithelium were observed to have extended into the connective tissue. This suggested that the disruption of the connective tissue collagen fibres resulted in the extension of the epithelial cells into the connective tissue. Thus, Silva et al. [22] also reported that the mitotic activity of the epithelial cells in the stratum basale increased with the development of diabetes.

In 1998, Hillman et al. [23] reported that under pathological conditions such as gingival inflammation, the stratum corneum became thicker (hyperkeratosis), with frequent fissures and flap lift off. They also reported that, as a result of inflammation, cells with nuclear fragments appeared (parakeratosis), because the migration period of cells to the surface was not long enough to allow the total transformation of the cell content into keratin.

In 2012, Monea et al. [24] reported an accelerated mitosis in the basal epithelial layer, acanthosis in the spinocellular layer, and parakeratosis and hyperkeratosis in the superficial layer.

In the present study, hyperkeratosis and parakeratosis of the gingivae were observed to have become more evident in the diabetes group on day 21. Furthermore, collagen synthesis was observed to have become disrupted and vasodilatation of the capillaries was pronounced (Figure 2(D)–(F)). These findings complied with the previously reported findings explained above.

## Conclusions

The overall assessment of the findings obtained in the present study clearly demonstrated that diabetes may induce the development of periodontal inflammation and tissue breakdown. However, it should be noted that, in the present study, an animal model was used and experimental diabetes was induced. Nevertheless, the present study provides valuable information on an issue discussed worldwide, and shows that diabetes acts as a factor, which aggravates periodontal disease. The results of the present study demonstrate that diabetes alone may cause alterations in the periodontium and affect periodontal health.

## Conflict of Interest

The authors declare that there is no conflict of interest regarding the publication of this paper.
